# Trigeminal nerve electrical stimulation attenuates early traumatic brain injury through the TLR4/NF-κB/NLRP3 signaling pathway mediated by orexin-A/OX1R system

**DOI:** 10.18632/aging.205795

**Published:** 2024-05-06

**Authors:** Junwei Kang, Yifan Zhou, Qi Xiong, Xiaoyang Dong

**Affiliations:** 1 Department of Rehabilitation Medicine, The First Affiliated Hospital, Jiangxi Medical College, Nanchang University, Nanchang 330006, Jiangxi, P.R. China

**Keywords:** trigeminal nerve electrical stimulation, traumatic brain injury, inflammation, TLR4/NF-κB/NLRP3 pathway, OX-A/OX1R

## Abstract

Background: Traumatic brain injury (TBI) is a significant contributor to global mortality and disability, and emerging evidence indicates that trigeminal nerve electrical stimulation (TNS) is a promising therapeutic intervention for neurological impairment following TBI. However, the precise mechanisms underlying the neuroprotective effects of TNS in TBI are poorly understood. Thus, the objective of this study was to investigate the potential involvement of the orexin-A (OX-A)/orexin receptor 1 (OX1R) mediated TLR4/NF-κB/NLRP3 signaling pathway in the neuroprotective effects of TNS in rats with TBI.

Methods: Sprague-Dawley rats were randomly assigned to four groups: sham, TBI, TBI+TNS+SB334867, and TBI+TNS. TBI was induced using a modified Feeney’s method, and subsequent behavioral assessments were conducted to evaluate neurological function. The trigeminal nerve trunk was isolated, and TNS was administered following the establishment of the TBI model. The levels of neuroinflammation, brain tissue damage, and proteins associated with the OX1R/TLR4/NF-κB/NLRP3 signaling pathway were assessed using hematoxylin-eosin staining, Nissl staining, western blot analysis, quantitative real-time polymerase chain reaction, and immunofluorescence techniques.

Results: The findings of our study indicate that TNS effectively mitigated tissue damage, reduced brain edema, and alleviated neurological deficits in rats with TBI. Furthermore, TNS demonstrated the ability to attenuate neuroinflammation levels and inhibit the expression of proteins associated with the TLR4/NF-κB/NLRP3 signaling pathway. However, it is important to note that the aforementioned effects of TNS were reversible upon intracerebroventricular injection of an OX1R antagonist.

Conclusion: TNS may prevent brain damage and relieve neurological deficits after a TBI by inhibiting inflammation, possibly via the TLR4/NF-κB/NLRP3 signaling pathway mediated by OX-A/OX1R.

## INTRODUCTION

Traumatic brain injury (TBI) significantly contributes to global morbidity and mortality [1]. It affects a substantial number of individuals, with over 1.7 million cases reported worldwide and 50,000 deaths in the United States alone [2]. In China, the incidence rate is approximately 13 cases per 100,000 people, imposing a considerable burden on both the society and the families of affected patients [3]. In addition, TBI can cause severe complications, including disorders of consciousness and cognitive impairment, which not only influence disease progression and prognosis but also profoundly impact the quality of life experienced by patients [4, 5]. TBI encompasses both primary and secondary injuries, with primary injuries resulting from mechanical forces and causing irreversible neural damage to the affected areas [6]. Consequently, it is crucial to mitigate secondary brain injuries such as neuroinflammation, autophagy, excitotoxicity, apoptosis, and oxidative stress during the early stages [7, 8]. Mounting evidence suggests that neuroinflammation plays a pivotal role in the pathophysiological mechanisms of TBI and may exacerbate secondary brain tissue damage or worsen the overall outcome of injury [9, 10]. Hence, amelioration of neuroinflammation can potentially enhance outcomes in individuals with TBI.

Neuroinflammation is a prominent cellular and molecular characteristic of the central nervous system that responds to diverse forms of injury. The pathways of toll-like receptor 4 (TLR4), nuclear factor-κB (NF-κB), and nucleotide-binding domain-like receptor protein 3 (NLRP3) play a role in the inflammatory response following brain injury [11]. Activation of TLR4 due to TBI can trigger the secretion of inflammatory cytokines, including interleukin-1β (IL-1β), tumor necrosis factor-α (TNF-α), and interleukin-18 (IL-18), through the NF-κB signaling pathway [11, 12]. Moreover, NF-κB can potentially enhance the expression of members of the NLR family, including the NLRP3 inflammasome. The NLRP3 inflammasome is a complex consisting of the innate immune receptor protein NLRP3, adapter protein apoptosis-associated speck-like protein (ASC), and inflammatory protease caspase-1 [13]. It is currently one of the most extensively studied inflammasomes and has been implicated in the promotion of proinflammatory cytokine production. Additionally, the NLRP3 inflammasome has been linked to various neurological disorders, such as ischemic stroke [14], Huntington’s [15] and Alzheimer’s disease [16]. Therefore, it is plausible to consider the NLRP3 inflammasome as a prospective therapeutic target for impeding the advancement of neuroinflammation induced by TBI.

There are currently alternative treatment options for TBI induced neurological deficits, including pharmacological treatment, hyperbaric oxygen therapy and neuromodulation [17, 18]. Neuromodulation, which includes both non-invasive and invasive brain stimulation techniques, exhibits substantial promise as a therapeutic option for a range of neurological disorders after brain damage [19, 20]. Previous studies have demonstrated that trigeminal nerve electrical stimulation (TNS) may have a neuroprotective effect in TBI and potentially upregulate the expression of hypocretin (also known as orexin-A) [21]. Orexin-A (OX-A) is synthesized in the lateral hypothalamus and regulates various physiological functions such as sleep/wake cycles, feeding, metabolic capacity, and neuroendocrine processes [22]. In addition, OX-A may inhibit NLRP3 activation to achieve neuroprotective outcomes [23, 24]. However, the precise mechanisms underlying the effects of TNS on TBI are poorly understood. Therefore, this study aims to assess the impact of TNS on rats with TBI and explore the potential association between the OX-A/OX1R mediated TLR4/NF-κB/NLRP3 signaling pathway.

## MATERIALS AND METHODS

### Animals and experimental groups

**Figure 1:**
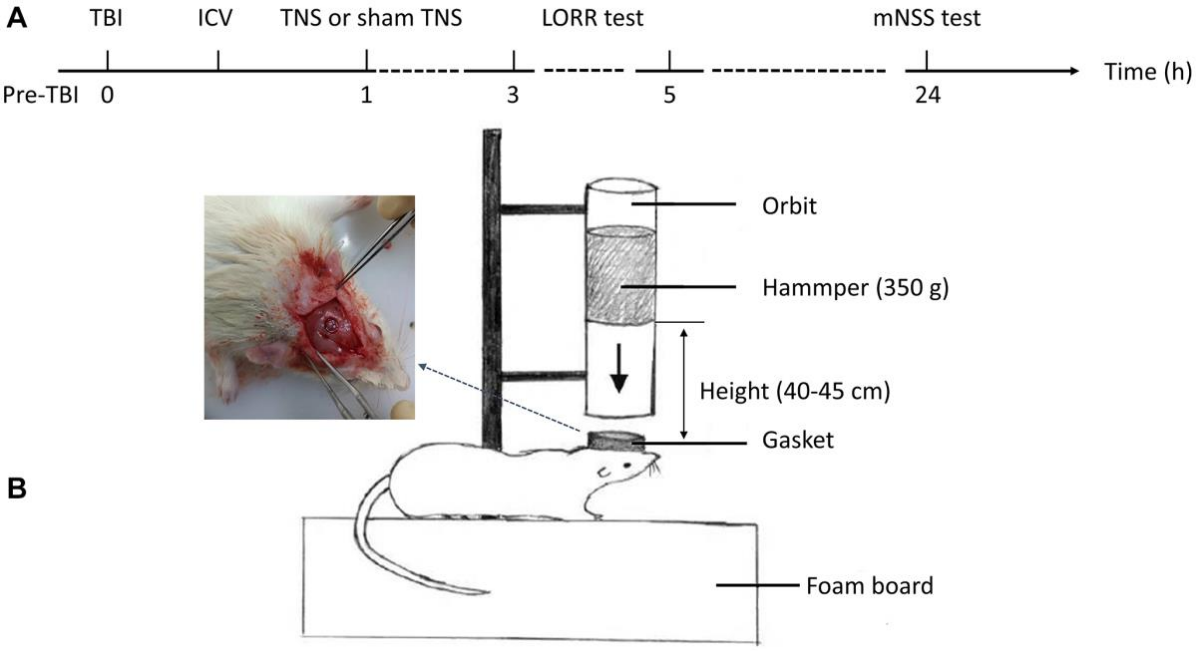
**Experimental flow and a model of traumatic brain injury (TBI).** (**A**) The schematic diagram depicted the intervention procedures and subsequent behavioral tests. (**B**) The construction of the TBI model was conducted in rats. Abbreviations: ICV: intracerebroventricular injection; TNS: trigeminal nerve electrical stimulation; LORR: loss of righting reflex; mNSS: modified neurological severity scale.

The Specific Pathogen Free (SPF) male adult Sprague-Dawley rats used in this study were sourced from the Nanchang Institute of Experimental Zoology (Nanchang, China) and were selected based on a body weight of 250–300 grams at 7 weeks of age. During their stay at the Nanchang University Experimental Animal Center, the rats were exposed to a 12-h cycle of day and night, room temperature 25°C, humidity is 50%, and high temperature sterilized water is used for drinking water. Additionally, the diet was specific for SPF rats. To ensure adaptation to laboratory conditions, the animals underwent a 7-day acclimation period prior to the commencement of the experiment. The rats were randomly assigned to four groups: sham (*n* = 18), which underwent sham surgery using dimethyl sulfoxide (DMSO); TBI (*n* = 18), which received DMSO and sham TNS after TBI; TBI+TNS (*n* = 18), which received DMSO and TNS after TBI; and TBI+TNS+SB334867 (*n* = 18), which received the OX1R antagonist SB334867 and TNS after TBI. The study protocol was approved by the Experimental Animal Welfare Ethics Committee of the First Affiliated Hospital of Nanchang University (approval number: CDYFY-IACUC-202302QR070) and was conducted in accordance with the guidelines for the Care and Use of Laboratory Animals provided by the National Institutes of Health. A schematic illustration of the experimental procedure is shown in Figure 1A.

### TBI model

The TBI rat model was established using a modified Feeney’s method as previously described [25] (Figure 1B). Initially, the animals were anesthetized with pentobarbital sodium (50 mg/kg, intraperitoneal injection) and their heads were secured in a stereotactic frame. A constant-temperature heating pad was used to maintain the body temperature. Following disinfection of the surgical area, a median incision approximately 10 mm in length was made on the scalp, and the skin and periosteum were carefully removed. Subsequently, a round bone window with a diameter of 5 mm was drilled using a cranial drill located 3 mm posterior to the coronal suture and 2.5 mm to the left side of the midline. The cranial disk was meticulously extracted without damaging the dura mater. Subsequently, a T-shaped lance was externally placed adjacent to the dura mater, followed by the release of a 40 g weight from a height of 20 cm along the guide tube. This action impacted the T-shaped lance, resulting in a 2–3 mm compression depth without breaching the dura mater, thereby inducing localized brain contusion and laceration. Immediately after impact, the T-shaped lance was withdrawn from the injury site to prevent secondary harm. Following the surgical procedure, the rats were housed in a temperature-controlled environment to facilitate post-anesthetic recovery and maintain physiological homeostasis.

### TNS

The TNS procedure was conducted 1 h after the TBI procedure. Unconscious rats were placed in the prone position during surgical intervention. A curvilinear supraorbital incision measuring 2 cm was made, allowing gentle lateral retraction of the orbital contents until the anterior ethmoidal nerve became visible. Subsequently, the ophthalmic nerves (V1) were isolated and the hook electrodes were positioned directly beneath the nerve. An ohmmeter was used to verify the ideal contact between the V1 nerve and the electrode. The probes of the ohmmeter were connected to both the electrical stimulator and electrode; subsequently, the electrode was securely attached to the V1 nerve. The electrode used in the experiment consisted of a pair of Teflon-coated silver hooks. To avoid any possible short circuits, the water was meticulously dried using a gauze. Subsequently, the electrodes were connected to an electrical stimulator (ES-420; ITO Physiotherapy and Rehabilitation) and the same procedure was repeated on the opposite side. Rats in the TBI+TNS groups were subjected to 30 min of bilateral electrical stimulation, whereas the TBI group did not receive any output electrical stimulation. The stimulation parameters included a frequency of 140 Hz, intensity of 1 mA, pulse width of 0.5 ms, duty cycle of 10 s on and 5 s off, and a duration of 30 min [21].

### Intracerebroventricular injection (ICV)

In a controlled environment, an injection catheter was inserted into the left cerebral ventricle of each rat 30 min after TBI. Prior to the surgical procedure, gentamicin, 0.1 mg/100 g body weight was intramuscularly injected into each rat. Rats were positioned within a stereotaxic frame (ZS-B/S; Beijing Zhongshi Dichuang Science and Technology Development Co., Ltd., Beijing, China) to ensure accurate placement of the guide cannula. The coordinates used for placement were as follows: 1.0 mm posterior to the bregma, 1.5 mm lateral to the midline, and 4.5 mm ventral to the surface of the skull. The incisor bar was positioned 3.2 mm below the interauricular line. Under sterile conditions, a cerebral ventricular injection catheter was inserted into each rat in the antagonist group. The OX1R inhibitor SB334867 (Tocris Bioscience, Ellisville, MO, USA) was dissolved in a 60:40 dimethyl sulfoxide solution and administered at a dose of 10 mg/kg body weight in a total volume of 5 μL. Following recovery from anesthesia, the rats underwent TNS.

### Loss of righting reflex test (LORR)

The LORR was assessed following TBI. An animal was deemed to have experienced an LORR if it was unable to correct its posture in the supine position. The duration of LORR was determined by measuring the time elapsed between the manifestation of LORR and subsequent recovery of the righting reflex, which served as criteria for the initiation and conclusion of hypnosis, respectively.

### Modified neurological severity scale (mNSS)

A 24-h evaluation of neurological function was conducted using the mNSS following the head injury. This test encompasses 10 distinct components that evaluate the motor (muscle status and abnormal movement), sensory (visual, tactile, and proprioceptive), balance, and reflex capabilities of rats. Scores for rats ranged from 0 to 18, with 0 indicating normal function and 18, the most severe deficit. We assigned one point to each behavior that was abnormal or the absence of a reflex that was tested. Two investigators who were blinded to the experimental groups conducted the neurobehavioral assessments. In the event of any discrepancy, a third researcher was consulted to reach a consensus.

### Brain water content

After completion of the experiment, the rats were euthanized by decapitation. Six brain tissue samples were collected from each group and divided into two halves along the midline. Subsequently, the cerebellum, meninges, lower brain stem, and olfactory bulb were dissected, rinsed with normal saline solution, and dried using filter paper. Once the samples were weighed in the glass vials, their initial wet weight was determined. Following this, a 24-h oven setting at 100°C was then used to heat the glass vials with samples. Subsequently, a second weight was measured to determine the dry weight of the samples. The formula for calculating brain water content is brain water content (%) = (wet weight - dry weight)/wet weight × 100%.

### Hematoxylin-eosin staining (H&E)

At the 24-h mark after the injury, six rats were decapitated under anesthesia induced by ether. After collecting brain tissues, they were fixed in 4% paraformaldehyde for 24 h at room temperature. The fixed samples were dehydrated using a series of graded alcohol concentrations ranging from 70 to 100% ethanol. Finally, the tissues were embedded in paraffin and sliced into sections with a thickness of 5 μm. Subsequently, the paraffin-embedded brain tissues were stained with H&E. In summary, sections were dewaxed and rehydrated using graded ethanol, followed by a 6-min hematoxylin staining and a 3-min eosin staining. The slides were then examined under a light microscope.

### Nissl staining

The paraffin-embedded tissue sections were dewaxed and hydrated. Subsequently, the sections were stained with Nishil’s dye (toluidine blue) for 5 min and rinsed thrice with distilled water for 20 s each. Xylene was applied for 5 min to the resulting brain slices, followed by dehydration using varying ethanol concentrations. Finally, the sections were sealed with neutral gum and examined under a light microscope.

### Western blot

Proteins were extracted from the pericontusive cortex (width, 3 mm; depth, 3 mm; length, 5 mm) and quantified using a Pierce BCA Protein Assay Kit (Thermo Fisher Scientific, Waltham, MA, USA). Western blotting was performed to quantify the expression of OX1R, TLR4, NF-p65, NLRP3, ASC, and Caspase-1. Bicinchoninic acid (Cat. no. PA115; Tiangen Biotech, China) was used to measure protein concentrations in the tissue lysates. The primary antibodies used for western blotting included anti-OX1R (1:1000, ab224368, Abcam, UK), anti-TLR4 (1:1000, ab13556, Abcam), anti-p65 (1:1000, D14E12, Cell Signaling Technology, USA), anti-NLRP3 (1:500, ERP23094-1, Abcam), anti-caspase-1 (1:500, ab207802, Abcam), anti-ASC (1:1000, ab283684, Abcam), anti-GAPDH (1:2000, 60004-1-lg, Proteintech, China), and anti-HDAC1 (1:2000, ab109411, Abcam). Subsequently, proteins were electrophoretically separated on sodium dodecyl sulfate-polyacrylamide gels and transferred on to polyvinylidene fluoride membranes. Secondary antibodies (1:5000, Zsgb-Bio, China) conjugated to horseradish peroxidase were incubated with the membranes for 60 min at ambient temperature to facilitate primary antibody binding. After western blotting, the Image Lab Software (version 3.0) was used to evaluate the results.

### Immunofluorescence staining

Immunofluorescence staining was employed on paraffin-embedded sections to evaluate TNF-α, IL-6, and IL-1β protein levels. The sections were prepared by cutting them to a thickness of 5 μm, followed by dewaxing in xylene and dehydration in a series of ethanol concentrations (100, 95, 85, and 80%). Subsequently, the sections were subjected to antigen retrieval using EDTA solution in a hot water bath for 30 min. After cooling, the slices were immersed in phosphate-buffered saline PBS (pH 7.4) for three cycles of five min each. After drying, bovine serum albumin (BSA; G5001, Servicebio, China) was sealed for 30 min. Subsequently, the sections were subjected to incubation overnight with anti-TNF-α (1:500, ab307164, Abcam), anti-IL-6 (1:50, ab233706, Abcam) and anti-IL-1β (1:50, ab283818, Abcam) at a temperature of 4°C. Next, a secondary antibody (1:300, Gb21303, Servicebio) was added after the sections were washed and decolorized in PBS (PH 7.3). The covered sections were incubated in the dark at an ambient temperature for 50 min. DAPI (G1012, Servicebio) solution was added after decolorization in PBS (pH 7.4), and incubated for 10 min at room temperature in the dark. The slides were then rinsed with water for 10 min, and a self-quenching agent was added for 5 min. The slides were examined under a light microscope.

### Quantitative real-time polymerase chain reaction (qRT-PCR)

Total RNA was extracted from the pericontusion-traumatic brain tissue using TRIzol reagent (Invitrogen, Waltham, MA, USA). An ultraviolet spectrophotometer (Shanghai Precision Scientific Instrument Corp., China) was used to determine RNA concentration. Subsequently, the extracted RNA was subjected to reverse transcription to synthesize cDNA using a commercial kit (TransGen Biotech, Beijing, China), following the manufacturer’s instructions. Amplification was conducted using the StepOne Real-Time PCR System (Thermo Fisher Scientific) and SYBR Green PCR Master Mix (TransGen Biotech). The experimental protocol involved denaturation at a temperature of 95°C for 15 min, followed by a series of 40 cycles that included annealing at 95°C for 10 s and extension at 60°C for 32 s. Subsequently, a melting curve analysis was performed, starting at 95°C for 15 s, followed by 60°C for 1 min, and concluding with 15 s at 95°C. The quantification of mRNA expression was carried out using the 2^−ΔΔCt^ method. The primers used in this study are listed in Table 1.

**Table 1:** The primer sequences for PCR amplification.

**Gene**	**Accession number**	**Primer sequence (5′–3′)**		**Tm (°C)**	**Product size (bp)**
R-IL-1β	NM_031512.2	Sense	CCCTTGACTTGGGCTGT	64.2	60
		Antisense	CGAGATGCTGCTGTGAGA	64.2	
R-IL-6	NM_012589.2	Sense	CACCAGGAACGAAAGTCAA	62.6	104
		Antisense	CAACAACATCAGTCCCAAGA	62.7	
R-TNF-α	NM_012675.3	Sense	CAGCCAGGAGGGAGAAC	63.9	93
		Antisense	GTATGAGAGGGACGGAACC	63.9	
R-GAPDH	NM_017008.4	Sense	TCTTTGCTTGGGTGGGT	63.7	148
		Antisense	TGGGTCTGGCATTGTTCT	63.7	

Abbreviations: PCR: polymerase chain reaction; Tm: melting temperature; GAPDH: glyceraldehyde 3-phosphate dehydrogenase; IL-1β: interleukin-1β; TNF-α: tumor necrosis factor-α; IL-6: interleukin-6.

### Statistical analyses

The normality of continuous variables was assessed using the Shapiro-Wilk test and the Levene test was used to do the Homogeneity of variance test. Normally distributed data were analyzed using analysis of variance, and non-normally distributed data were analyzed using the Kruskal-Wallis test. Statistical analyses were performed using GraphPad Prism 9.0 (GraphPad Software, San Diego, CA, USA). The statistical outcomes are presented as mean ± standard deviation (SD), with a significance level of *P* < 0.05.

## RESULTS

### TNS reduced neurological deficits, tissue damage, and brain water content in TBI models

**Figure 2:**
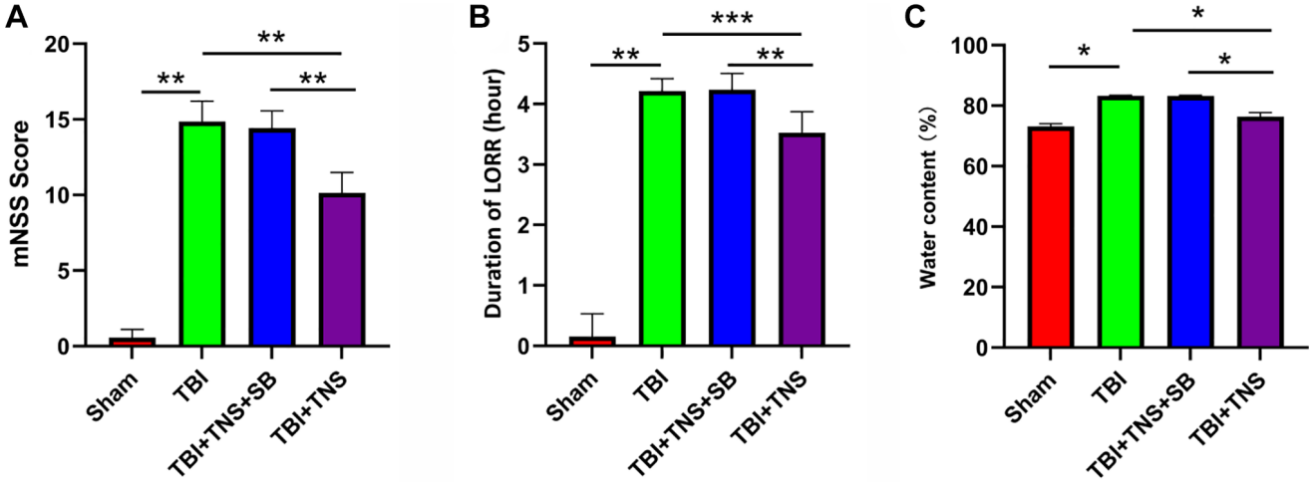
**Trigeminal nerve electrical stimulation (TNS) mitigates neurological deficits and cerebral edema.** (**A**, **B**) Neurological function was analyzed and modified by neurological severity scores (mNSS) and loss of righting reflex test (LORR) after traumatic brain injury (TBI). (**C**) The brain water content of each group was detected 24 h post-TBI (^*^P < 0.05, ^**^P < 0.01, ^***^P < 0.001).

The LORR and mNSS tests were conducted in rats to assess the impact of TNS on neurological function following TBI. The results indicated that the TBI group exhibited elevated mNSS scores and prolonged LORR duration compared with the sham group. However, the administration of TNS significantly reduced the mNSS scores and shortened the duration of LORR, suggesting that TNS treatment facilitated neurological recovery after TBI (Figure 2A, 2B).

**Figure 3:**
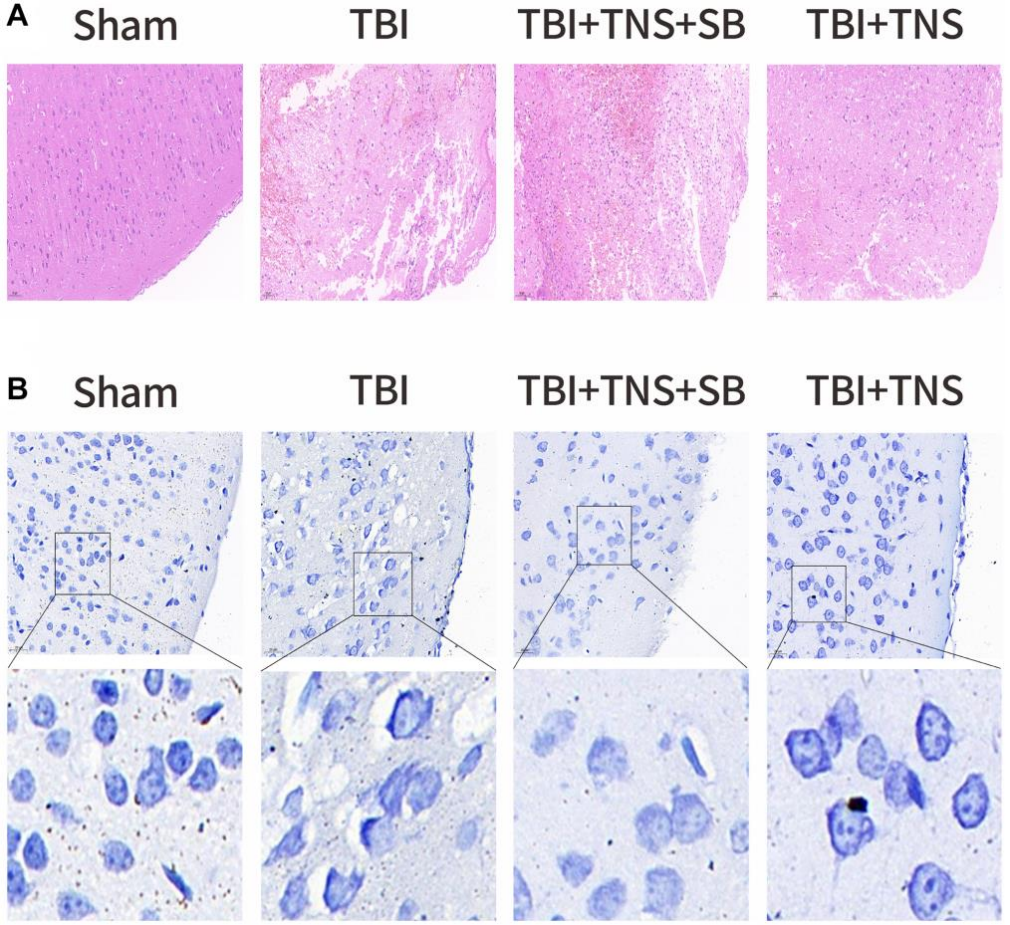
**Trigeminal nerve electrical stimulation (TNS) relieves tissue damage and neuronal degeneration.** (**A**) Representative hematoxylin-eosin staining images of four groups of cortical tissues showing areas of necrosis, scale = 50 μm. (**B**) Representative Nissl staining of four groups of cortical tissues demonstrates necrotic degeneration of neurons, scale = 20 μm.

We conducted additional investigations to examine the therapeutic effects of TNS on TBI-induced brain edema and tissue damage. Brain water content in the TBI group was significantly higher than that in the sham group. However, administration of TNS significantly improved brain edema, as depicted in Figure 2C. Histological analyses using H&E and Nissl staining demonstrated that the sham group exhibited well-defined and compact brain structures with normal and uniformly arranged neuronal structures. In contrast, the TBI group exhibited loosened structures, extensive vacuolar changes, and neuronal degeneration. However, administration of TNS significantly improved these conditions, as shown in Figure 3A, 3B. Consequently, TNS treatment significantly mitigated brain edema and tissue damage in affected brain tissues.

### TNS upregulation of OX-A/OX1R expression and inhibition of inflammation in TBI models

**Figure 4:**
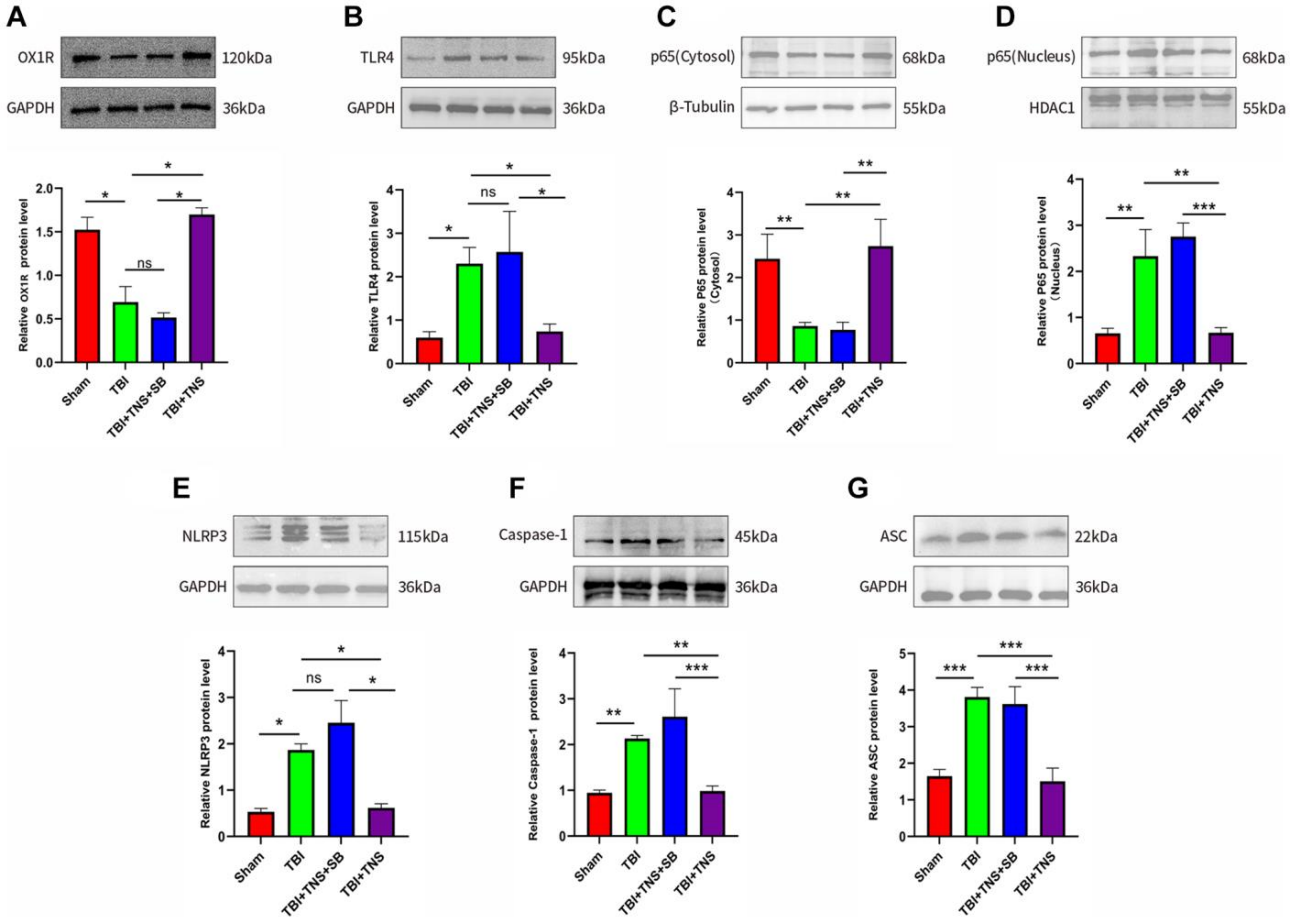
**Trigeminal nerve electrical stimulation (TNS) inhibits toll-like receptor 4 (TLR4)/NF-κB (nuclear factor kappa B)/nucleotide-binding domain (NOD)-like receptor protein 3 (NLRP3) pathway after traumatic brain injury (TBI) via orexin-A (OX-A)/orexin receptor 1 (OX1R).** (**A**) Protein levels of OX1R. (**B**) Protein levels of TLR4. (**C**) NF-κB (p65) protein level in the cytosol. (**D**) NF-κB (p65) protein level in the nucleus. (**E**) Protein levels of NLRP3. (**F**) Protein levels of caspase-1. (**G**) Protein levels of apoptosis-associated speck-like protein (ASC). Results are expressed as mean ± standard deviation (SD; ^*^P < 0.05, ^**^P < 0.01, ^***^P < 0.001).

The OX1R content in the tissues was quantified by western blotting to assess the impact of TNS on OX1R expression. Western blot results indicated that OX1R expression in the brain tissue of the TBI group was lower than that in the sham group (*P* < 0.05) (Figure 4A). However, the concentration of OX1R in the TBI+TNS group was significantly higher than that in the TBI group (Figure 4A), suggesting that TNS can potentially enhance the expression of OX1R.

**Figure 5:**
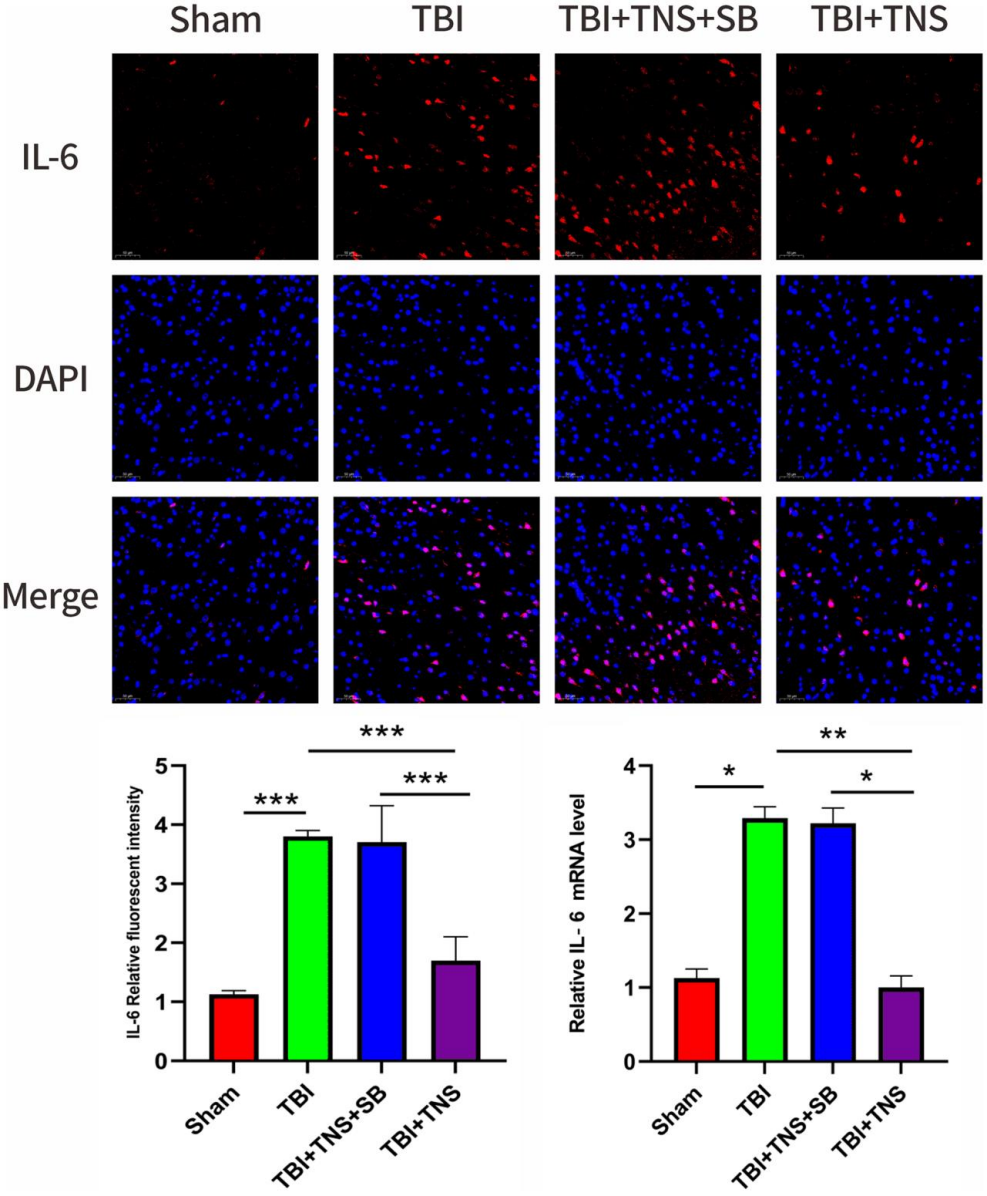
**Trigeminal nerve electrical stimulation (TNS) decreases interleukin (IL)-6 levels after traumatic brain injury (TBI) via orexin-A (OX-A)/orexin receptor 1 (OX1R).** Representative immunofluorescence staining and relative mRNA levels of interleukin IL-6. Results are expressed as mean ± standard deviation (SD; ^*^P < 0.05, ^**^P < 0.01, ^***^P < 0.001).

**Figure 6:**
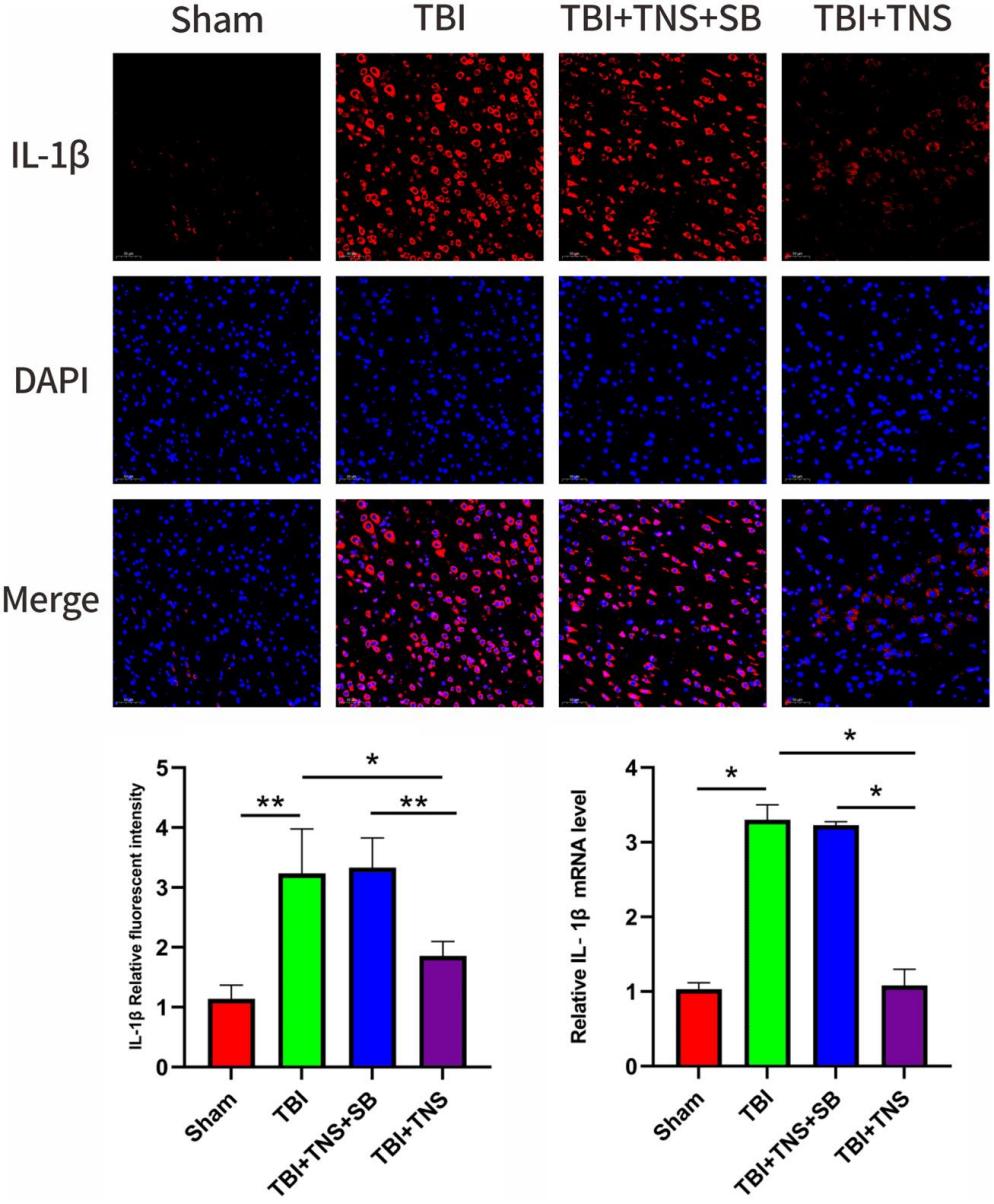
**Trigeminal nerve electrical stimulation (TNS) decreases interleukin (IL)-1β levels after traumatic brain injury (TBI) via orexin-A (OX-A)/orexin receptor 1 (OX1R).** Representative immunofluorescence staining and relative mRNA levels of IL-1β. Results are expressed as mean ± standard deviation (SD; ^*^P < 0.05, ^**^P < 0.01, ^***^P < 0.001).

**Figure 7:**
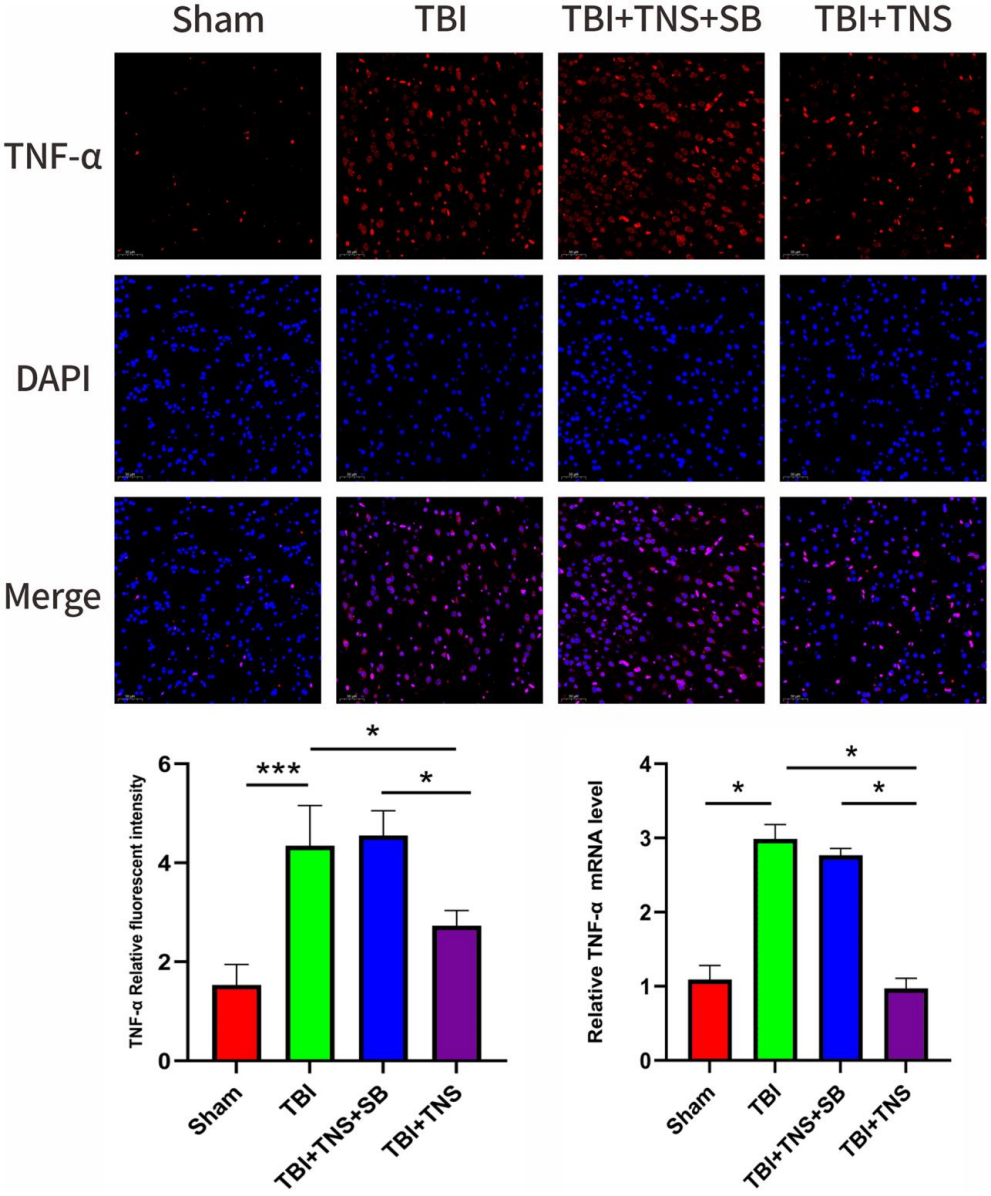
**Trigeminal nerve electrical stimulation (TNS) decreases tumor necrosis factor (TNF)-α levels after traumatic brain injury (TBI) via orexin-A (OX-A)/orexin receptor 1 (OX1R).** Representative immunofluorescence staining and relative mRNA levels of TNF-α. Results are expressed as mean ± standard deviation (SD; ^*^P < 0.05, ^**^P < 0.01, ^***^P < 0.001).

The inflammatory response following TBI plays a crucial role in developing neurological impairment. Additionally, we observed the effect of TNS on inflammation in a TBI model. Immunofluorescence staining revealed that the IL-1β, IL-6, and TNF-α levels matched with their corresponding mRNA levels (Figures 5–7). Compared to the sham group, the TBI group exhibited significantly elevated levels of IL-1β, IL-6, and TNF-α. In contrast, the TBI+TNS group demonstrated reduced levels of these proinflammatory factors compared to the TBI group. However, the effectiveness of TNS was negated when SB334867 was used to inhibit OX-A/OX1R function.

### TNS inhibited the activation of TLR4/NF-κB/NLRP3 inflammasome via OX-A/OX1R in TBI models

The TLR4/NF-κB/NLRP3 signaling pathway plays is pivotal in the inflammatory response following TBI. The protein expression of TLR4 was significantly higher in the TBI group than in the sham group, whereas it was lower in the TBI+TNS group than in the TBI group (Figure 4B). Additionally, TBI significantly decreased the protein level of NF-kB (p65) in the nucleus and increased it in the cytosol, but these changes were reversed with TNS treatment (Figure 4C, 4D), suggesting that TNS inhibits the translocation of NF-κB from the cytoplasm to the nucleus. Following TBI and subsequent treatment with TNS, protein levels of the NLRP3 inflammasome, including NLRP3, ASC, and caspase-1, were significantly altered (Figure 4E–4G). Specifically, the protein levels of NLRP3, caspase-1, and ASC were significantly increased in the TBI group, but decreased after TNS intervention. However, the aforementioned effects of TNS were attenuated following intracerebroventricular administration of the OX-A/OX1R antagonist, SB334867. These findings suggest that TNS may impede the activation of the TLR4/NF-KB/NLRP3 inflammatory pathway through OX-A/OX1R signaling.

## DISCUSSION

**Figure 8:**
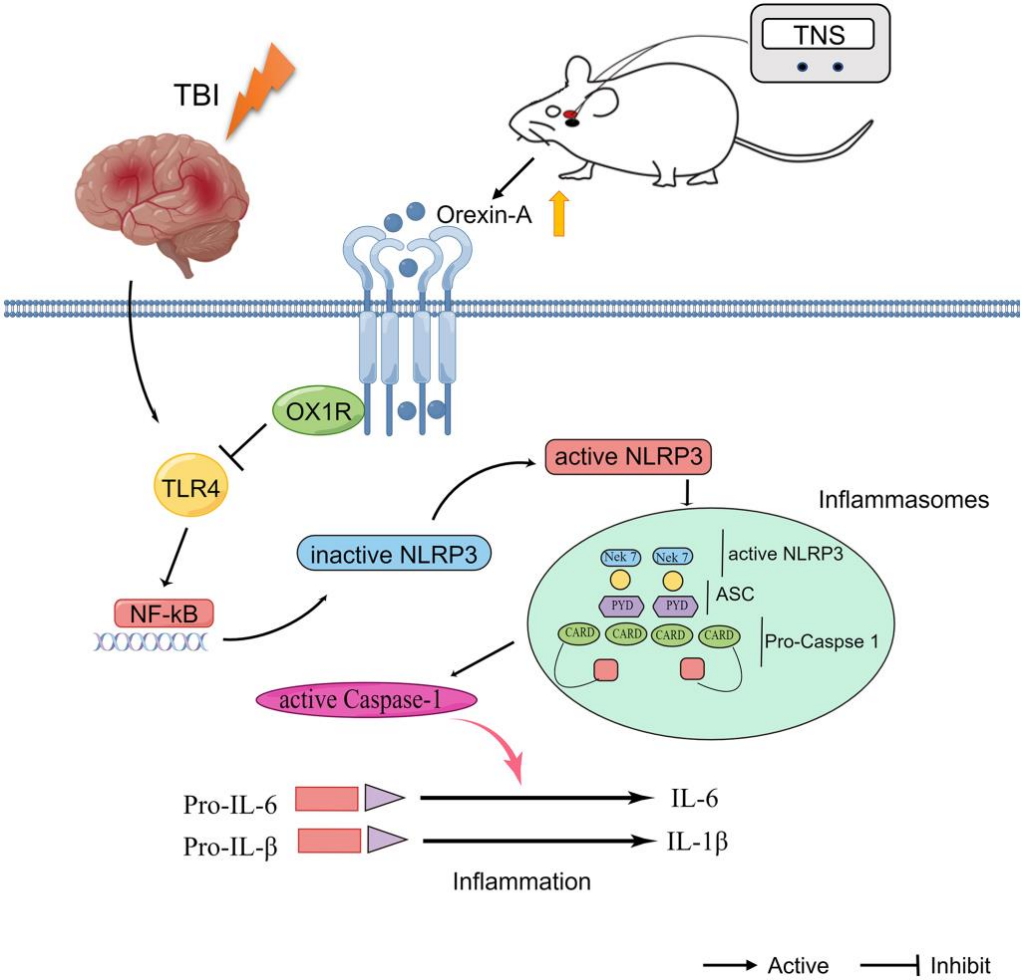
**Schematic diagram depicting the potential mechanism and protective effects of trigeminal nerve electrical stimulation (TNS) on traumatic brain injury (TBI).** TNS exerts neuroprotective effects in TBI model rats through inhibition of inflammation mediated by orexin-A (OX-A)/orexin receptor 1 (OX1R)/toll-like receptor 4 (TLR4)/NF-κB (nuclear factor kappa B)/nucleotide-binding domain (NOD)-like receptor protein 3 (NLRP3) signaling pathway.

In this study, we observed that TNS effectively mitigated neurological disorders in rats with TBI by suppressing inflammatory responses. Furthermore, it was discovered that TNS significantly hindered the TLR4/NF-κB/NLRP3 signaling pathway in the region of brain injury, and this effect could be reversed by the administration of an OX-A/OX1R antagonist, SB334867. The findings of this study provide evidence that TNS may contribute to neuroprotection in a model of TBI by modulating the TLR4/NF-κB/NLRP3 signaling pathway via OX-A/OX1R, as illustrated in Figure 8.

TNS is a novel neuromodulation technique that has been used for the therapeutic management of various neurological conditions, including epilepsy, depression, disorders of consciousness, and cognitive impairment [26–28]. Our previous investigation yielded preliminary evidence suggesting that TNS holds promise as a viable and secure intervention to restore consciousness in individuals with neurological disorders [29]. More recently, a randomized, double-blind sham-controlled study demonstrated that TNS could potentially to enhance local brain metabolism and facilitate functional recuperation in patients with prolonged disorders of consciousness [28].

Multiple studies have provided evidence of the neuroprotective effects of TNS in animal models of TBI. Chiluwal et al. found that TNS significantly reduced brain edema, blood-brain barrier disruption, lesion volume, and brain cortical levels of TNF-α and IL-6 in TBI rats [30]. Similar findings were observed in animal behavior tests, where TNS not only protected the blood-brain barrier and reduced brain edema, but also potentially influenced the TNF-α and IL-6 levels, as well as downregulated the cleaved caspase-3 signaling pathway [31]. Furthermore, Zheng et al. discovered that TNS has the potential to enhance the consciousness level and EEG activity of TBI rats by activating the neuronal activity of OX-A and the spinal trigeminal nucleus [21]. In the current investigation, we observed that TNS can reduce the duration of LORR and decrease the scores of mNSS, as well as alleviate brain edema and downregulate the expression of IL-1β, IL-6, and TNF-α in TBI rats. However, the molecular mechanisms underlying the neuroprotective effects of TNS on TBI remain poorly understood.

Neuroinflammation is a pivotal cellular and molecular characteristic of the central nervous system (CNS) in response to various insults, such as trauma [32, 33]. Microglia, which are indigenous innate immune cells within the CNS, are recognized as agents responsible for mediating the neuroinflammatory response that ensues after TBI. The activation of these cells triggers a plethora of inflammatory cascades, encompassing the generation and liberation of subsequent proinflammatory cytokines, namely IL-1β and IL-6 [34, 35]. Inflammasomes and elevated levels of inflammasome proteins during the acute phase following TBI are significant contributors to the pathological inflammatory response, and these increased levels have been linked to unfavorable functional outcomes [36]. Our study aligns with prior research, indicating that IL-1β, IL-6, and TNF-α levels were significantly enhanced and correlated with diminished performance following TBI. NOD-like receptors (NLRs) are an important group of innate immune system regulators. The NLR is a cytosolic pattern recognition receptor based on three components: an adaptor protein, a sensor molecule, and an effector protein. Upon activation, these individual units assemble to create an inflammasome, which is a complex with pro-inflammatory properties. Among the various NLRs found in mammals, NLRP3 has been extensively investigated [10]. Our findings demonstrate the upregulation of NLRP3 inflammasome expression in rats with TBI.

The TLR4/NF-κB signaling pathway is widely recognized as a canonical pathway responsible for activating the NLRP3 inflammasome [37, 38]. The NLRP3 inflammasome plays a critical role in diseases associated with inflammation by promoting the transcription of proinflammatory cytokine genes and establishing a positive feedback loop. In this study, we observed that the TLR4/NF-κB signaling pathway triggered the activation of NLRP3 following TBI, resulting in neurological deficits. However, these symptoms were significantly ameliorated by the TNS treatment. Furthermore, we conducted additional investigations to elucidate the mechanisms through which TNS modulates the TLR4/NF-κB/NLRP3 signaling pathway.

OX-A is a neuropeptide that exhibits neuroprotective effects by reducing apoptosis, inflammation, and oxidative stress [39, 40]. Our previous study demonstrated that vagus nerve stimulation facilitates the restoration of consciousness in TBI models by upregulating the expression of OX-A/OX1R [41]. Similarly, Zheng et al. discovered that transcutaneous electrical nerve stimulation is an effective therapeutic approach for managing unconsciousness by activating neurons in the lateral hypothalamus and upregulating OX-A expression [21]. Consistently, our study also observed activation of OX-A/OX1R following transcutaneous electrical nerve stimulation intervention. Moreover, empirical data indicate that OX-A can potentially mitigate astrocytic apoptosis and inflammation by activating NF-κB signaling pathways in models of cerebral ischemia/reperfusion injury [42]. In the present investigation, we have discovered that TNS exhibits the ability to suppress the TLR4/NF-κB/NLRP3 signaling pathway, and this effect can be counteracted by the administration of the OX1R antagonist, SB334867. The combined results of these studies demonstrate that TNS exerts its anti-inflammatory effects by inhibiting the TLR4/NF-κB/NLRP3 cascade via the OX-A/OX1R pathway.

This study had several limitations. First, our investigation focused solely on the neuroprotective effects of the TNS in the early stages of brain injury following TBI. Further research is required to examine the effects of TNS during the chronic stages of brain damage after a TBI, including chronic cognitive impairment and prolonged disorders of consciousness. Second, the TNS parameters utilized in this study were solely derived from previous studies, necessitating the exploration and comparison of different stimulation parameters to ascertain the optimal parameters for neuroprotection following TBI. Finally, TBI may initiate a variety of pathological processes; however, in our study, we focused on the inflammatory response following TBI. Hence, the efficacy of TNS in exerting its neuroprotective effects via alternative pathways remains unclear. Furthermore, future investigations should focus on elucidating the mechanisms underlying TNS.

## CONCLUSIONS

In conclusion, the findings of this study provide evidence that TNS facilitates neurological recovery and mitigates brain injury in TBI by suppressing inflammation mediated by the TLR4/NF-κB/NLRP3 signaling pathway through OX-A/OX1R. These results offer valuable insights into the potential therapeutic applications of TNS for TBI management.
